# Low-dose CT for quantitative analysis in acute respiratory distress syndrome

**DOI:** 10.1186/cc12866

**Published:** 2013-08-31

**Authors:** Vittoria Vecchi, Thomas Langer, Massimo Bellomi, Cristiano Rampinelli, Kevin K Chung, Leopoldo C Cancio, Luciano Gattinoni, Andriy I Batchinsky

**Affiliations:** 1 Comprehensive Intensive Care Research Task Area, US Army Institute of Surgical Research, 3698 Chambers Pass, Fort Sam Houston, TX, 78234-7767, USA; 2 School of Medicine, Università degli Studi di Milano, via Festa del Perdono 7, 20122 Milan, Italy; 3 National Research Council, National Academies, 500 Fifth Street, NW, Washington, DC, 20001, USA; 4 Dipartimento di Fisiopatologia medico-chirurgica e dei trapianti, Università degli Studi di Milano, via Francesco Sforza, 35, 20122 Milan, Italy; 5 Dipartimento di Radiologia, Istituto Europeo di Oncologia, via Ripamonti 435, 20141 Milan, Italy; 6 Dipartimento di Anestesia, Rianimazione (Intensiva e Subintensiva) e Terapia del Dolore, Fondazione IRCCS Ca' Granda - Ospedale Maggiore Policlinico, via Francesco Sforza, 35, 20122 Milan, Italy

## Abstract

**Introduction:**

The clinical use of serial quantitative computed tomography (CT) to characterize lung disease and guide the optimization of mechanical ventilation in patients with acute respiratory distress syndrome (ARDS) is limited by the risk of cumulative radiation exposure and by the difficulties and risks related to transferring patients to the CT room. We evaluated the effects of tube current-time product (mAs) variations on quantitative results in healthy lungs and in experimental ARDS in order to support the use of low-dose CT for quantitative analysis.

**Methods:**

In 14 sheep chest CT was performed at baseline and after the induction of ARDS via intravenous oleic acid injection. For each CT session, two consecutive scans were obtained applying two different mAs: 60 mAs was paired with 140, 15 or 7.5 mAs. All other CT parameters were kept unaltered (tube voltage 120 kVp, collimation 32 × 0.5 mm, pitch 0.85, matrix 512 × 512, pixel size 0.625 × 0.625 mm). Quantitative results obtained at different mAs were compared via Bland-Altman analysis.

**Results:**

Good agreement was observed between 60 mAs and 140 mAs and between 60 mAs and 15 mAs (all biases less than 1%). A further reduction of mAs to 7.5 mAs caused an increase in the bias of poorly aerated and nonaerated tissue (-2.9% and 2.4%, respectively) and determined a significant widening of the limits of agreement for the same compartments (-10.5% to 4.8% for poorly aerated tissue and -5.9% to 10.8% for nonaerated tissue). Estimated mean effective dose at 140, 60, 15 and 7.5 mAs corresponded to 17.8, 7.4, 2.0 and 0.9 mSv, respectively. Image noise of scans performed at 140, 60, 15 and 7.5 mAs corresponded to 10, 16, 38 and 74 Hounsfield units, respectively.

**Conclusions:**

A reduction of effective dose up to 70% has been achieved with minimal effects on lung quantitative results. Low-dose computed tomography provides accurate quantitative results and could be used to characterize lung compartment distribution and possibly monitor time-course of ARDS with a lower risk of exposure to ionizing radiation. A further radiation dose reduction is associated with lower accuracy in quantitative results.

## Introduction

Chest computed tomography (CT) and the related lung quantitative CT (qCT) analysis have greatly improved the understanding of the pathophysiological and morphological features of acute respiratory distress syndrome (ARDS) [1-6]. Moreover, qCT has been proposed as a valuable tool to determine the potential for lung recruitment (thus optimizing the setting of positive end-expiratory pressure [7]) and to assess lung opening and closing as well as lung hyperinflation in the effort to reduce the occurrence of ventilator-induced lung injury [8,9].

Besides the difficulties and risks related to transferring patients to the CT room, one of the major factors hindering the adoption of serial qCT is the associated patient exposure to ionizing radiation [10-12]. Radiation dose is linearly related to the tube current-exposure time product (mAs) which affects the image noise level and thus influences image quality [13]. In general, an increase in mAs will improve image quality at the cost of a higher radiation dose, while a reduction in mAs will have the opposite effect [14], but other factors may be implied, such as tissue-weighting, use of automatic tube current modulation technique or variations in peak kilovoltage (kVp) between others.

It is worth mentioning that, despite the extensive use of qCT, a standardized protocol for the acquisition parameters of CT images has not been defined, and, in particular, widely variable mAs have been reported in both experimental [15,16] and clinical settings [17,18]. Although low-dose CT has been used extensively in other fields [19-21], limited data are available on its application for lung quantitative analysis. Indeed, aside from a few studies on pulmonary emphysema [22-24] that showed that quantification of hyperinflated tissue is not affected by a reduction of tube current-exposure time product to 20 mAs [22], no data are available on the possible effects of different mAs values on quantitative lung analysis results.

If quantitative results performed on low- to ultra-low-dose chest CT scans were accurate, qCT could be used more frequently to characterize lung compartment distribution and potential for lung recruitment with reduced radiation exposure. The aim of the present study was therefore to investigate the effects of variations in mAs on quantitative results in healthy lungs and in experimental ARDS.

## Materials and methods

This study was approved by the US Army Institute of Surgical Research Animal Care and Use Committee and was conducted in compliance with the Animal Welfare Act, the implementing Animal Welfare Regulations and the principles of the Guide for the Care and Use of Laboratory Animals.

Fourteen anesthetized and mechanically ventilated female sheep (44 ± 6 kg, one to two years of age) were studied. All animals were included in other protocols conducted at the US Army Institute of Surgical Research (that is, no animal was used for the sole purpose of this study). Further details are provided in the Supplementary Material.

### Computed tomography scan image acquisition and reconstruction

Chest CT (Aquilion 64, Toshiba America Medical Systems, Tustin, CA, USA) was performed at baseline (healthy lungs) and six to eight hours after the induction of ARDS. Experimental ARDS was induced via intravenous injection of 0.1 to 0.15 ml/kg oleic acid [25].

Before CT scanning, the degree of inflation of the cuff of the endotracheal or tracheostomy tube was checked to minimize or avoid the possible air leakage. During CT image acquisition, two consecutive scans were obtained after having clamped the endotracheal or tracheostomy tube during a respiratory hold performed with the mechanical ventilator (Servo 300; Siemens, Solna, Sweden). The entire lung was imaged. For each couple of scans, two different mAs were applied in randomized order to compare the corresponding quantitative results: 60 mAs was chosen as the reference value according to the weight range of the studied animals [26,27] and was paired with 140, 15 or 7.5 mAs. Each couple of scans acquired at the same airway pressure during a respiratory hold therefore consisted of a scan performed at 60 mAs and a scan performed at 140, 15 or 7.5 mAs. All other CT parameters were kept unaltered (tube voltage 120 kVp, rotation time 0.5 s, collimation 32 × 0.5 mm, pitch 0.85, reconstruction matrix 512 × 512, pixel size 0.625 × 0.625 mm). An automatic tube current modulation technique was not applied during scan acquisition. Images were reconstructed using a 5-mm section width, a 5-mm interval and a body standard axial filter (FC13).

### Quantitative analysis

Images were processed using image analysis software (Maluna 3.17; Göttingen, Germany). The pulmonary tissue was selected as previously described [28]. Briefly, lung boundaries were drawn automatically on each baseline image and manually on each CT image of sheep with experimental ARDS. After processing each slice of a series, total lung volume, total lung tissue mass and frequency distribution of lung CT numbers expressed in Hounsfield units (HUs) were computed. Based on their degree of aeration, four different lung compartments were quantified according to usual thresholds [3]: hyperinflated tissue (-1,000 to -901 HU), normally aerated tissue (-900 to -501 HU), poorly aerated tissue (-500 to -101 HU) and nonaerated tissue (-100 to +200 HU).

### Dose and noise evaluation

The volumetric computed tomography dose index (CTDI_vol_) and dose-length product (DLP) of each scan were provided by the CT scanner. Effective dose (E) was estimated using the DLP method [29]. Image noise levels for each applied mAs were calculated as the mean standard deviation (SD) of tissue density in a uniform area (within the aorta) of 10 different scans [30].

### Statistical analysis

Data are expressed as mean ± SD unless otherwise stated. Results obtained at baseline and after the induction of ARDS were analyzed separately. The agreement between quantitative results obtained from consecutive scans performed with different mAs was assessed using Bland-Altman analysis [31], linear regression and paired *t*-test or signed rank-sum test as appropriate. The difference between CT number frequency distribution of the different mAs was assessed by paired *t*-test or signed rank-sum test as appropriate. One-way analysis of variance was used to compare CTDI_vol_, DLP, E and image noise of the different applied mAs. A rank transformation was used for non-normally distributed variables that did not pass the equal variance test. Statistical significance was defined as *P *< 0.05. Statistical analysis was performed with SigmaPlot 11.2 software (Systat, Chicago, IL, USA).

## Results

A total of 218 CT scans were acquired, 92 at baseline and 126 during experimental ARDS. Forty comparisons between 60 and 140 mAs (12 at baseline and 28 during experimental ARDS), 36 comparisons between 60 and 15 mAs (18 at baseline and 18 during experimental ARDS) and 33 comparisons between 60 and 7.5 mAs (16 at baseline and 17 during experimental ARDS) were performed.

**Figure 1:**
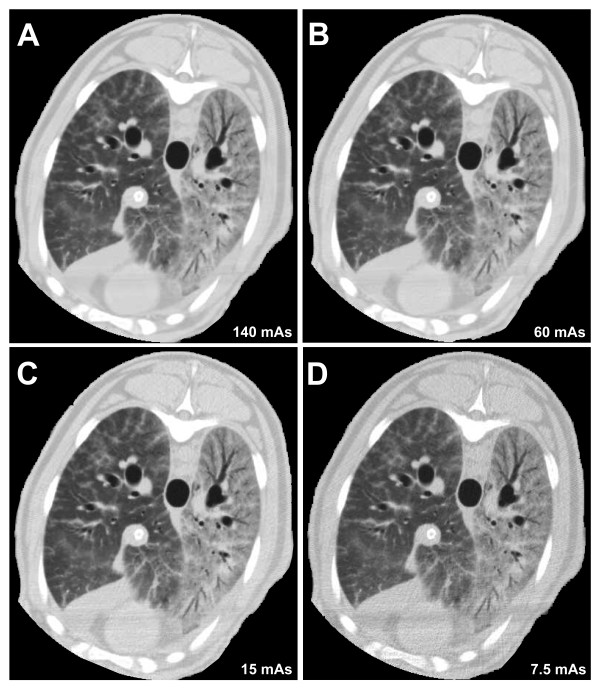
**Lung computed tomography images of a sheep with acute respiratory distress syndrome induced by oleic acid**. These images show the change in image quality due to the different tube current-exposure time products (mAs) applied. **(A) **140 mAs. **(B) **60 mAs. **(C) **15 mAs. **(D) **7.5 mAs. Despite increased image noise, the interface between lung and surrounding structures can easily be recognized.

The reduction of mAs was associated with an increase in image noise and a worsening of image quality (Figure 1). However, the increased image noise did not hinder the recognition of the interface between lung and surrounding structures.

**Figure 2:**
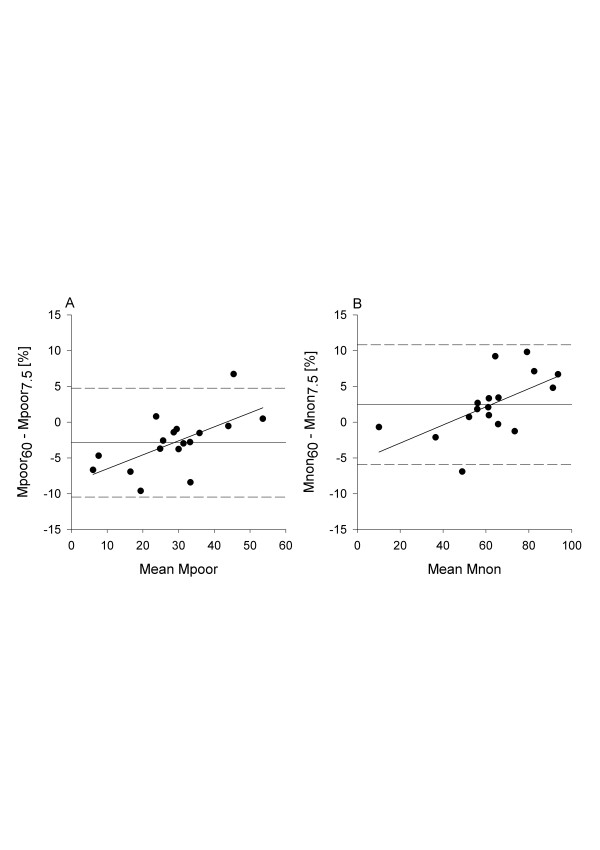
**Bland-Altman analysis of poorly aerated and nonaerated lung tissue for computed tomography scans performed at 60 and 7.5 tube current-exposure time products after the induction of acute respiratory distress syndrome**. All masses are expressed as percentages of total lung mass of tissue. Values on the *x*-axis represent the average of values recorded with two tube current-exposure time products (mAs), for example, mean M_poor _= (M_poor _CT_60 _+ M_poor _CT_7.5_)/2. **(A) **Poorly aerated mass: slope = 0.20, *r*^2 ^= 0.39, *P *= 0.01. **(B) **Nonaerated mass: slope = 0.13, *r*^2 ^= 0.35, *P *= 0.01. CT = computed tomography; M_poor _= poorly aerated mass; M_non _= nonaerated mass.

Both in healthy lungs and during experimental ARDS, excellent agreement was observed between qCT results obtained at 60 and 140 mAs (Table 1), as well as good agreement between those obtained at 60 mAs and 15 mAs (Table 2). The further reduction of current-exposure time product to 7.5 mAs was associated with a marked increase of bias and limits of agreement of Bland-Altman analysis, in particular for poorly aerated and nonaerated lung compartments of sheep with experimental ARDS (Table 3 and Figure 2). Additional Bland-Altman plots of different comparisons are reported in the Supplementary Material (Additional file 1, Figures E1 to E6).

**Table 1:** Comparison between quantitative computed tomography results obtained at 60 and 140 tube current-exposure time product^a^

60 mAs to 140 mAs		Mean ± SD_60_	Mean ± SD_140_	*P*	*r* ^2^	Bias	LOA
**Baseline (*n *= 12)**	Lung volume (ml)	2,782 ± 666	2,785 ± 671	0.88	0.99	1.7	-114.0 to 109.0
	Lung tissue mass (g)	679 ± 102	683 ± 101	0.12	1.00	-4.5	-13.2 to 4.2
	Hyperinflated tissue (%)	0.6 ± 1.1	0.5 ± 1.0	0.08	0.97	0.1	-0.4 to 0.5
	Normally aerated tissue (%)	86.6 ± 2.9	86.4 ± 3.2	0.52	0.95	0.2	-1.7 to 2.1
	Poorly aerated tissue (%)	11.5 ± 3.4	11.8 ± 3.7	0.21	0.95	-0.3	-2.0 to 1.3
	Nonaerated tissue (%)	1.4 ± 0.5	1.3 ± 0.7	0.07	0.98	0.1	-0.1 to 0.2

**ARDS (*n *= 28)**	Lung volume (ml)	2,022 ± 338	2,023 ± 343	0.91	0.99	-0.7	-64.3 to 62.8
	Lung tissue mass (g)	1,500 ± 159	1,498 ± 151	0.73	0.98	1.8	-51.4 to 55.0
	Hyperinflated tissue (%)	0.0 ± 0.1	0.0 ± 0.1	0.52	0.95	0.0	-0.1 to 0.1
	Normally aerated tissue (%)	11.4 ± 11.1	11.4 ± 11.1	0.91	0.99	0.0	-2.4 to 2.4
	Poorly aerated tissue (%)	33.1 ± 15.6	33.2 ± 16.0	0.72	0.98	0.0	-4.5 to 4.4
	Nonaerated tissue (%)	55.4 ± 23.2	55.4 ± 23.5	0.99	0.99	0.0	-5.5 to 5.5

^a^ARDS = acute respiratory distress syndrome; mAs = tube current-exposure time product; lung volume = total lung volume; lung tissue mass = total mass of lung tissue; hyperinflated tissue = mass of hyperinflated tissue; normally aerated tissue = mass of normally aerated tissue; poorly aerated tissue = mass of poorly aerated tissue; nonaerated tissue = mass of nonaerated tissue; *P *= *P*-value of the comparison between values obtained at 60 and at 140 mAs by paired *t*-test or signed rank-sum test as appropriate; *r*^2 ^= coefficient of determination of linear regression between values obtained at 60 and 140 mAs; bias and LOA = bias and limits of agreement (bias ± 1.96 SD) of the Bland-Altman analysis.

**Table 2:** Comparison between quantitative computed tomography results obtained at 60 and 15 tube current-exposure time product^a^

60 mAs to 15 mAs		Mean ± SD_60_	Mean ± SD_15_	*P*	*r* ^2^	Bias	LOA
**Baseline (*n *= 18)**	Lung volume (ml)	3,227 ± 1,015	3,224 ± 995	0.80	1.0	3.4	-112.0 to 118.8
	Lung tissue mass (g)	711 ± 128	707 ± 129	0.17	0.99	4.3	-21.8 to 30.4
	Hyperinflated tissue (%)	3.3 ± 5.5	3.9 ± 5.6	<0.001	0.99	-0.7	-1.8 to 0.5
	Normally aerated tissue (%)	81.4 ± 7.5	81.1 ± 6.6	0.22	0.93	0.3	-4.2 to 4.8
	Poorly aerated tissue (%)	13.3 ± 6.2	12.9 ± 6.2	0.84	0.91	0.4	-3.3 to 4.1
	Non aerated tissue (%)	2.1 ± 1.4	2.1 ± 1.5	0.04	0.96	-0.1	-0.6 to 0.5

**ARDS (*n *= 18)**	Lung volume (ml)	2,295 ± 561	2,264 ± 526	0.07	0.99	31.4	-105.8 to 168.6
	Lung tissue mass (g)	1,778 ± 315	1,775 ± 301	0.78	0.98	3.0	-85.1 to 91.1
	Hyperinflated tissue (%)	0.1 ± 0.2	0.2 ± 0.2	0.008	0.94	0.0	-0.1 to 0.1
	Normally aerated tissue (%)	9.3 ± 9.7	8.8 ± 9.4	0.13	0.98	0.5	-2.1 to 3.2
	Poorly aerated tissue (%)	28.1 ± 13.4	28.6 ± 12.6	0.44	0.96	-0.5	-4.8 to 3.9
	Non aerated tissue (%)	62.5 ± 21.2	62.5 ± 20.0	0.98	0.98	0.0	-6.9 to 6.9

^a^ARDS = acute respiratory distress syndrome; mAs = tube current-exposure time product; lung volume = total lung volume; lung tissue mass = total mass of lung tissue; hyperinflated tissue = mass of hyperinflated tissue; normally aerated tissue = mass of normally aerated tissue; poorly aerated tissue = mass of poorly aerated tissue; nonaerated tissue = mass of nonaerated tissue; *P *= *P*-value of the comparison between values obtained at 60 and at 15 mAs by paired *t*-test or signed rank-sum test as appropriate; *r*^2 ^= coefficient of determination of linear regression between values obtained at 60 and 15 mAs; bias and LOA = bias and limits of agreement (bias ± 1.96 SD) of the Bland-Altman analysis.

**Table 3:** Comparison between quantitative computed tomography results obtained at 60 and 7.5 tube current-exposure time product^a^

60 mAs to 7.5 mAs		Mean ± SD_60_	Mean ± SD_7.5_	*P*	*r* ^2^	Bias	LOA
**Baseline (*n *= 16)**	Lung volume (ml)	3,180 ± 1,096	3,162 ± 1,083	0.18	1.0	17.7	-87.9 to 123.3
	Lung tissue mass (g)	726 ± 106	716 ± 111	0.12	0.96	9.8	-39.2 to 58.8
	Hyperinflated tissue (%)	2.7 ± 4.0	4.2 ± 4.7	<0.001	0.94	-1.5	-4.1 to 1.1
	Normally aerated tissue (%)	81.9 ± 7.1	79.6 ± 5.6	0.002	0.90	2.3	-2.9 to 7.6
	Poorly aerated tissue (%)	13.4 ± 6.9	14.1 ± 6.4	0.50	0.88	-0.7	-5.4 to 3.9
	Nonaerated tissue (%)	2.1 ± 1.4	2.2 ± 1.3	0.09	0.98	-0.1	-0.5 to 0.3

**ARDS (*n *= 17)**	Lung volume (ml)	2,321 ± 499	2,281 ± 478	0.01	0.99	40.4	-73.8 to 154.6
	Lung tissue mass (g)	1,807± 286	1,797 ± 295	0.41	0.97	10.0	-86.1 to 106.1
	Hyperinflated tissue (%)	0.0 ± 0.0	0.1 ±0.0	0.004	0.79	0.0	-0.1 to 0.0
	Normally aerated tissue (%)	9.1 ± 9.5	8.7 ± 9.1	0.20	0.98	0.4	-2.1 to 3.0
	Poorly aerated tissue (%)	27.3 ± 13.7	30.2 ± 11.3	0.008	0.95	-2.9	-10.5 to 4.8
	Nonaerated tissue (%)	63.5 ± 21.4	61.1 ± 18.8	0.03	0.97	2.4	-5.9 to 10.8

^a^ARDS = acute respiratory distress syndrome; mAs = tube current-exposure time product; lung volume = total lung volume; lung tissue mass = total mass of lung tissue; hyperinflated tissue = mass of hyperinflated tissue; normally aerated tissue = mass of normally aerated tissue; poorly aerated tissue = mass of poorly aerated tissue; nonaerated tissue = mass of nonaerated tissue; *P *= *P*-value of the comparison between values obtained at 60 and at 7.5 mAs by paired *t*-test or signed rank-sum test as appropriate; *r*^2 ^= coefficient of determination of linear regression between values obtained at 60 and 7.5 mAs; bias and LOA = bias and limits of agreement (bias ± 1.96 SD) of the Bland-Altman analysis.

**Figure 3:**
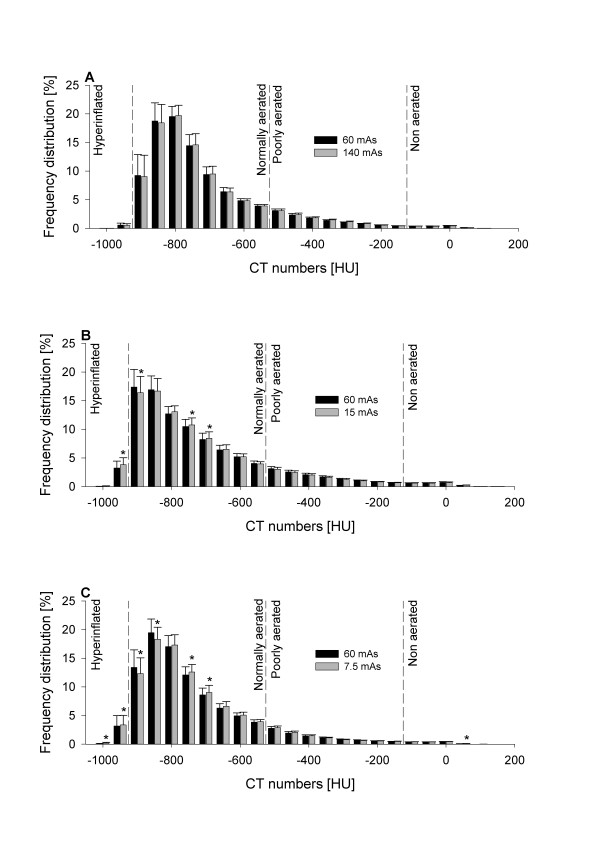
**Mean frequency distribution of CT numbers of scans performed at baseline (healthy lungs) expressed as percentages of tissue mass and grouped into intervals of 50 HU**. Data are presented as mean ± SE. **(A) **Comparison between 60 and 140 tube current-exposure time products (mAs). **(B) **Comparison between 60 and 15 mAs. **(C) **Comparison between 60 and 7.5 mAs. *P < 0.05 vs. 60 mAs by paired *t*-test or rank-sum test as appropriate. Vertical dashed lines delimit lung compartments as defined in Materials and methods. CT = computed tomography; HU = Hounsfield units.

**Figure 4:**
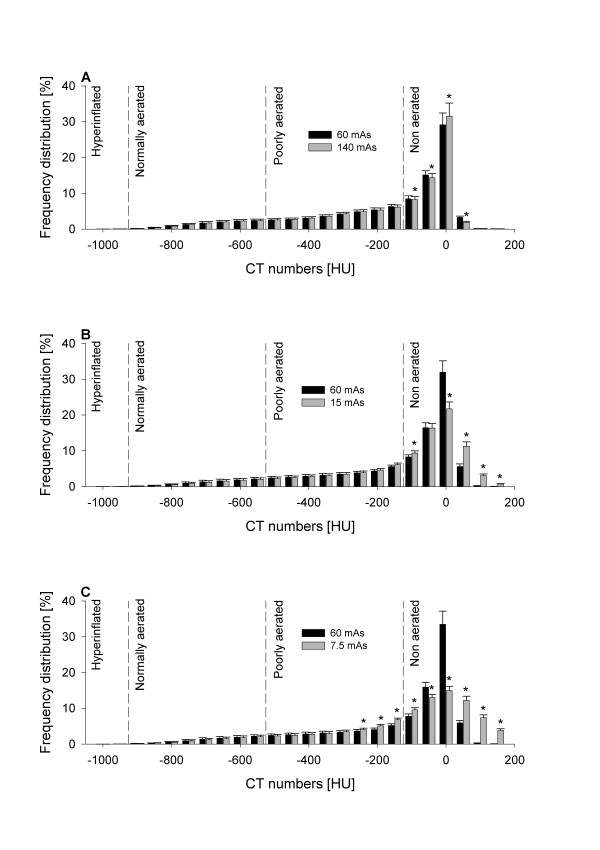
**Mean frequency distribution of CT numbers of scans performed on sheep with experimental acute respiratory distress syndrome expressed as percentages of tissue mass and grouped into intervals of 50 HU**. Data are presented as mean ± SE. **(A) **Comparison between 60 and 140 tube current-exposure time products (mAs). **(B) **Comparison between 60 and 15 mAs. **(C) **Comparison between 60 and 7.5 mAs. *P < 0.05 vs. 60 mAs by paired *t*-test or rank-sum test as appropriate. Vertical dashed lines delimit lung compartments as defined in Materials and methods. CT = computed tomography; HU = Hounsfield units.

The frequency distributions of CT numbers at different mAs in healthy sheep and sheep with experimental ARDS are reported in Figure 3 and Figure 4, respectively. Of note, the reduction of mAs to 7.5 mAs caused significant changes in the frequency distribution of CT numbers.

Mean recorded values of CTDI_vol_, DLP, image noise and mean estimated value of E are reported in Table 4. When comparing the mean values of E at 15 mAs (2.0 ± 0.8 mSv) and at 7.5 mAs (0.9 ± 0.1 mSv) to the mean value of E at 60 mAs (7.4 ± 0.9 mSv), dose reductions of 73% and 88%, respectively, were achieved. Additional results are provided in the Supplementary Material.

**Table 4:** Dose and noise evaluation^a^

Measurement	140 mAs	60 mAs	15 mAs	7.5 mAs	*P*-value
CTDI_vol _(mGy)	22.1 ± 0.0	9.2 ± 0.8	2.2 ± 0.3	1.1 ± 0.1	<0.001
DLP (mGy/cm)	870.5 ± 47.7	362.2 ± 45.2	96.5 ± 39.0	44.9 ± 6.4	<0.001
E (mSv)	17.8 ± 1.0	7.4 ± 0.9	2.0 ± 0.8	0.9 ± 0.1	<0.001
Image noise (HU)	10.0 ± 1.1	15.9 ± 3.5	37.5 ± 10.6	73.8 ± 17.5	<0.001

^a^CTDI_vol _= volume computed tomography dose index; DLP = dose-length product; E = effective dose; HU = Hounsfield unit; Image noise = image noise of each applied mAs calculated as the mean SD of tissue density (expressed in HUs) of ten regions of interest placed on a uniform tissue (aorta) in ten different scans; mAs: tube current-exposure time product; mSv = millisievert; *P *= *P*-value of one-way analysis of variance. Data are expressed as mean ± SD.

## Discussion

In this study, we have shown that a reduction of effective dose up to 70% can be achieved with minimal effects on lung quantitative results and that low-dose CT could therefore be a valuable tool for the characterization of lung compartment distribution and possibly for monitoring the time-course of ARDS with a lower risk of exposure to ionizing radiation.

Quantitative results obtained at 60 mAs were compared with (1) the results obtained at a higher dose (140 mAs) chosen within the range of doses commonly used for standard chest CT in adults [32] and (2) the results obtained at two progressively lower doses (15 and 7.5 mAs).

We analyzed scans obtained from healthy sheep and from sheep with experimental ARDS. Overall, the majority of lung tissue (about 80%) was normally aerated at baseline, whereas approximately 90% of lung tissue was poorly aerated or nonaerated after the induction of ARDS.

On the one hand, by analysis of healthy lungs, we aimed to study the pure physical effects of mAs-related noise variations on the quantitative analysis. Indeed, the interface between healthy pulmonary parenchyma and surrounding structures (that is, thoracic wall, mediastinum, diaphragm, hilar vessels and main and lobar bronchi) was perfectly recognizable, regardless of the applied mAs, and the difference between their densities allowed the use of the automated function of the quantitative analysis software to outline the regions of interest. In this group, we can therefore safely state that, in the compared scans, equivalent regions of interest were analyzed.

On the other hand, when analyzing scans of injured sheep, the possibility of an additional effect had to be taken into account. Indeed, considering the similarity between densities of injured lungs and other thoracic structures, the operator-dependent ability to recognize lung boundaries could have been impaired by the worsening image quality (noisier) of the lower-dose images (15 and 7.5 mAs). This in turn could have led to differences in the manual selection of regions of interest within compared scans. In this regard, despite the slight change in image noise (Table 4), image quality did not vary notably between scans obtained at 60 and 140 mAs (Figures 1A and 1B). Moreover, although the significant increase in image noise caused a progressive deterioration in image quality, even on scans performed at 15 and 7.5 mAs (Figures 1C and 1D), the recognition of lung and surrounding structures (which is the sole requirement to perform qCT) was preserved.

Both in healthy lungs and in lungs with experimental ARDS, quantitative results obtained at 60 and 140 mAs showed excellent limits of agreement and biases close to 0%, and the statistical analysis did not indicate any significant difference (Table 1). Also, the comparison of qCT data obtained at 60 and 15 mAs (Table 2) showed good limits of agreement and biases lower than 1%. However, a further reduction of mAs to 7.5 caused both an increase in bias and a widening of the limits of agreement, especially for poorly aerated and nonaerated lung tissue in sheep with experimental ARDS.

Figures 3 and 4, besides illustrating the evident densitometric change between healthy and injured lungs, show that the mAs reduction increased image noise (Table 4) and thus caused a progressive change in frequency distribution of CT numbers. Indeed, the comparison of frequency distribution of CT numbers between 60 and 15 mAs and between 60 and 7.5 mAs at baseline (Figures 3B and 3C) showed a progressive shift of tissue from the normally inflated to the hyperinflated compartment that was related to the reduction in mAs. Similarly, when observing the comparison of frequency distribution of CT numbers between 60 and 15 mAs and between 60 and 7.5 mAs during experimental ARDS (Figures 4B and 4C), a progressive shift of tissue from the nonaerated to the poorly aerated compartment was measured. The change in frequency distribution also explains the significant decrease in total lung volume, which was observed especially at the lowest dose on scans obtained during experimental ARDS. Indeed, the observed widening of the frequency distribution of density at 7.5 mAs (Figure 4C) caused a shift of nonaerated tissue both toward the poorly aerated compartment (as described above) and toward CT numbers greater than the threshold of +200 HU, commonly used as the upper limit for nonaerated tissue. It is worth mentioning that, for this reason, tissue densities measured as greater than +200 HU were excluded from the overall computation, despite being part of the region of interest. This fact explains the underestimation at 7.5 mAs of total lung volume. Moreover, as part of nonaerated tissue is shifted toward CT numbers not included in the overall computation, this effect also accounts in part for the above-mentioned reduction of nonaerated tissue measured at 7.5 mAs. Of note, the underestimation of total lung volume and (in part) of nonaerated tissue could be avoided by increasing the included HU range (for example, up to +500 HU).

The described effect of image noise level on the frequency distribution of tissue density is also clearly represented when analyzing a region of interest positioned on a uniform tissue (aorta): the progressive reduction of mAs is associated with a lowering of the distribution peak and a corresponding widening of the distribution curve (See Additional File 1, Figure E7).

The establishment of a standardized protocol would prevent any mAs-related difference in quantitative results. Indeed, similarly to the reconstruction parameters [33], identical acquisition parameters need to be used to compare quantitative results of different scans performed on the same patient as well as quantitative results of different studies, scanners and institutions [34]. When defining a standardized CT acquisition protocol for quantitative analysis, the "ALARA concept" (As Low As Reasonably Achievable) [35,36] should be taken into account. Our study supports the use of low-dose CT for this purpose. Indeed, we may speculate that, at the same effective dose of one scan performed at 140 mAs, approximately two scans at 60 mAs or ten scans at 15 mAs could be performed (Table 4). Moreover, it is worth mentioning that the use of low-dose CT could be coupled with the simplified analysis method, based on the extrapolation of whole-lung results from ten CT scan slices [37,38]. This method, besides shortening the time needed to perform qCT, would allow a further reduction of radiation dose.

A limitation of the present study that we need to point out is that the absolute mAs values used in this experimental study cannot be applied directly to patients with ARDS. Indeed, being that image noise directly correlates to body weight [39], it is conceivable that such mAs values would be associated with higher image noise, which could therefore affect quantitative results significantly. Moreover, the worsening of image quality caused by a substantial reduction of mAs during CT acquisition should be kept in mind, as it could limit the diagnostic viability of CT examinations.

## Conclusions

A reduction of effective dose up to 70% can be achieved with minimal effects on lung quantitative results. Lung quantitative analysis performed on low-dose CT scans provides accurate results both in healthy lungs and in experimental ARDS; therefore, it is a valuable tool for characterizing and potentially monitoring lung disease. In particular, if multiple chest CT scans are performed to characterize the lung quantitatively and assess the response to the application of different airway pressures (potential for lung recruitment), low-dose CT could be used to reduce patients' radiation exposure. This, of course, needs to be proved in the real world of ICUs.

## Key messages

 • Quantitative lung analysis performed using low-dose CT scans is accurate.

• The effective dose can be reduced by up to 70% with minimal effects on quantitative results.

• If multiple chest CT scans are performed to characterize the lung quantitatively and assess the response to the application of different airway pressures (potential for lung recruitment), low-dose CT can be used to reduce the patient's radiation exposure.

• The use of ultra-low-dose CT increases image noise significantly and reduces the accuracy of lung quantitative analysis.

## Abbreviations

ARDS: Acute respiratory distress syndrome; CT: Computed tomography; CTDI_vol_: Volume computed tomography dose index; DLP: Dose-length product; E: Effective dose; HU: Hounsfield unit; kVp: Peak kilovoltage; mAs: Tube current-exposure time product; qCT: quantitative computed tomography; SD: standard deviation.

## Competing interests

The authors declare that they have no competing interests.

## Authors' contributions

VV, TL and AIB conceived the study, performed the experiments, analyzed the data and wrote the manuscript. VV and TL processed CT images and performed quantitative analysis. MB, CR, KKC, LCC and LG participated in study design and critically revised the manuscript. All authors read and approved the final version of the manuscript.

## Supplementary Material

Additional file 1 **Electronic Supplementary Material**. Additional Materials, Results and References Click here for file
