# Supplementary material for: Low-dose CT for quantitative analysis in acute respiratory distress syndrome
Source: Crit Care. 2013 Aug 31;17(4):R183. doi: 10.1186/cc12866 (PMC4057189; doi:10.1186/cc12866)
Supplement: Additional file 1 — Electronic Supplementary Material. Additional Materials, Results and References [file cc12866-S1.DOC]

**Low-dose CT for Quantitative Analysis in the Acute Respiratory Distress Syndrome**

Vittoria Vecchi, Thomas Langer, Massimo Bellomi, Cristiano Rampinelli,

Kevin K Chung, Leopoldo C Cancio, Luciano Gattinoni and Andriy I Batchinsky

***Electronic Supplementary Material***

The opinions or assertions contained herein are the private views of the authors, and are not to be construed as official or as reflecting the views of the Department of the Army or the Department of Defense.

**Additional methods**

*Anesthesia plan and veterinary intensive care unit care*

Anesthesia was induced with an intramuscular injection of tiletamine-zolazepam and buprenorphine followed by isoflurane 2-4% delivered via mask. Subsequently, sheep were intubated in the prone position with an 8 mm ID endotracheal tube and connected to the mechanical ventilator (Fabius, Dräger Medical, Lübeck, Germany). Propofol was injected intravenously to maintain anesthesia during CT image acquisition.

Between different CT sessions animals were housed in a dedicated veterinary intensive care unit with continuous animal monitoring performed by experienced technicians under the supervision of the primary investigators.

*Ventilatory settings during CT image acquisition*

Chest CT (Toshiba Aquilion 64-slice Medical System, Tustin, CA) was performed at baseline (healthy lungs) and 6-8 hours after the induction of ARDS either during an inspiratory hold (30 cmH_2_O) or during an expiratory hold (0 cmH_2_O) performed with the mechanical ventilator (Servo 300, Siemens, Solna, Sweden).

*CT-scan image analysis*

Hilar vessels, main and lobar bronchi were excluded from the selected region of interest. To avoid inter-observer variability [E1], each CT-couple was processed by the same operator (either VV or TL).

The total lung volume was calculated as follows:

$Lung volume _{[tot]}=Number of voxels \times Volume of voxel$ Eq. 1

Where “Lung volume _[tot]_” defines the total volume of the lung (expressed in milliliters), “Number of voxels” represents the total number of voxels included in the regions of interest and “Volume of voxel” represents the volume of the voxel (0.001953 ml).

The volumes of the differently aerated lung compartments (hyperinflated, normally aerated, poorly aerated and non aerated) were computed similarly:

$Lung volume _{[compartment]} =Number of voxels _{[compartment]} \times Volume of voxel$ Eq. 2

Where “Lung volume _[compartment]_” represents the volume of a specific compartment (expressed in milliliters) and “Number of voxels _[compartment]_” represents the number of voxels included in the considered compartment.

The total lung tissue mass was calculated as follows:

$Lung tissue mass _{[tot]}=Lung volume _{[tot]} \times\left(1-\frac{CT_{mean}}{-1000}\right)$ Eq. 3

Where “Lung tissue mass _[tot]_” represents the total mass of the lung (expressed in grams), “Lung volume _[tot]_” represents the total lung volume (expressed in milliliters) (see Equation 1) and “CT_mean_” represents the mean CT number of the whole lung (expressed in Hounsfield Units).

The mass of tissue of the four different lung compartments was computed as:

Eq. 4

Where “Tissue mass _[compartment]_” represents the mass of tissue of a specific compartment (expressed in grams), “Lung volume _[compartment]_” represents the lung volume of the considered compartment (see Equation 2) and “CT_mean [compartment]_” represents the mean CT number of the considered compartment (expressed in Hounsfield Units).

**Additional results**

**Figure E1.**

**Figure E1.** Bland-Altman analysis of CT scans performed at 60 and 140 mAs at baseline.

*Definition of abbreviations*: M_hyper_ = hyperinflated mass; M_norm_ = normally aerated mass; M_poor_ = poorly aerated mass; M_non_ = non aerated mass. All masses are expressed as percentage of total lung mass of tissue. Values on the X-axis represent the average between values recorded with two mAs, e.g., Mean Mhyper = (Mhyper CT_60_+Mhyper CT_140_)/2. Hyperinflated mass (**Panel A**): slope = 0.10, r^2^ = 0.24, p = 0.11. Normally aerated mass (**Panel B**): slope = -0.12, r^2^ = 0.09, p = 0.24. Poorly aerated mass (**Panel C)**: slope = -0.07, r^2^ = 0.00, p = 0.33. Non aerated mass (**Panel D)**: slope = -0.05, r^2^  = 0.09, p = 0.35.

**Figure E2.**

**Figure E2.** Bland-Altman analysis of CT scans performed at 60 and 140 mAs after the induction of experimental ARDS.

*Definition of abbreviations*: M_hyper_ = hyperinflated mass; M_norm_ = normally aerated mass; M_poor_ = poorly aerated mass; M_non_ = non aerated mass. All masses are expressed as percentage of total lung mass of tissue. Values on the X-axis represent the average between values recorded with two mAs, e.g., Mean Mhyper = (Mhyper CT_60_+Mhyper CT_140_)/2. Hyperinflated mass (**Panel A**): slope = -0.18, r^2^ = 0.21, p = 0.02. Normally aerated mass (**Panel B**): slope = 0.00, r^2^ = 0.00, p = 0.85. Poorly aerated mass (**Panel C)**: slope = -0.02, r^2^ = 0.03, p = 0.39. Non aerated mass (**Panel D)**: slope = -0.01, r^2^  = 0.01, p = 0.68.

**Figure E3.**

**Figure E3.** Bland-Altman analysis of CT scans performed at 60 and 15 mAs at baseline.

*Definition of abbreviations*: M_hyper_ = hyperinflated mass; M_norm_ = normally aerated mass; M_poor_ = poorly aerated mass; M_non_ = non aerated mass. All masses are expressed as percentage of total lung mass of tissue. Values on the X-axis represent the average between values recorded with two mAs, e.g., Mean Mhyper = (Mhyper CT_60_+Mhyper CT_15_)/2. Hyperinflated mass (**Panel A**): slope = -0.02, r^2^ = 0.03, p = 0.49. Normally aerated mass (**Panel B**): slope = 0.12, r^2^ = 0.14, p = 0.11. Poorly aerated mass (**Panel C)**: slope = 0.01, r^2^ = 0.00, p = 0.88. Non aerated mass (**Panel D)**: slope = -0.07, r^2^  = 0.12, p = 0.14.

**Figure E4.**

**Figure E4.** Bland-Altman analysis of CT scans performed at 60 and 15 mAs after the induction of experimental ARDS.

*Definition of abbreviations*: M_hyper_ = hyperinflated mass; M_norm_ = normally aerated mass; M_poor_ = poorly aerated mass; M_non_ = non aerated mass. All masses are expressed as percentage of total lung mass of tissue. Values on the X-axis represent the average between values recorded with two mAs, e.g., Mean Mhyper = (Mhyper CT_60_+Mhyper CT_15_)/2. Hyperinflated mass (**Panel A**): slope = -0.09, r^2^ = 0.11, p = 0.18. Normally aerated mass (**Panel B**): slope = 0.04, r^2^ = 0.06, p = 0.32. Poorly aerated mass (**Panel C)**: slope = 0.06, r^2^ = 0.08, p = 0.25. Non aerated mass (**Panel D)**: slope = 0.06, r^2^  = 0.13, p = 0.15.

**Figure E5.**

**Figure E5.** Bland-Altman analysis of CT scans performed at 60 and 7.5 mAs at baseline.

Definition of abbreviations: Mhyper = hyperinflated mass; Mnorm = normally aerated mass; Mpoor = poorly aerated mass; Mnon = non aerated mass. All masses are expressed as percentage of total lung mass of tissue. Values on the X-axis represent the average between values recorded with two mAs, e.g., Mean Mhyper = (Mhyper CT_60_+Mhyper CT_7.5_)/2. Hyperinflated mass (**Panel A**): slope = -0.17, r^2^ = 0.30, p = 0.02. Normally aerated mass (**Panel B**): slope = 0.24, r^2^ = 0.30, p = 0.02. Poorly aerated mass (**Panel C**): slope = 0.08, r^2^ = 0.05, p = 0.38. Non aerated mass (**Panel D**): slope = 0.08, r^2^ = 0.30, p = 0.02.

**Figure E6.**

**Figure E6.** Bland-Altman analysis of CT scans performed at 60 and 7.5 mAs after the induction of experimental ARDS.

*Definition of abbreviations*: M_hyper_ = hyperinflated mass; M_norm_ = normally aerated mass. All masses are expressed as percentage of total lung mass of tissue. Values on the X-axis represent the average between values recorded with two mAs, e.g., Mean Mhyper = (Mhyper CT_60_+Mhyper CT_7.5_)/2. Hyperinflated mass (**Panel A**): slope = -0.13, r^2^ = 0.06, p = 0.34. Normally aerated mass (**Panel B**): slope = 0.05, r^2^ = 0.11, p = 0.20. For Bland-Altman plots of poorly aerated mass and non aerated mass see **Figure 2** of the main manuscript.

**Figure E7.**

**Figure E7.** Comparison between frequency distributions of CT numbers of a homogenous tissue at 140, 60, 15 and 7.5 mAs. Each curve represents the mean distribution of ten regions of interest placed in the aorta of ten different scans. Standard deviations were not represented for clarity of the graph.

**Table E1. Gas Exchange at baseline and after the induction of experimental acute respiratory distress syndrome.**

| **Gas Exchange variables** | **Baseline** | **ARDS** |
| --- | --- | --- |
| pH | 7.43±0.04 | 7.30±0.08 |
| PCO_2_ [mmHg] | 39±3 | 45±9 |
| PO_2_ [mmHg] | 225±18 | 88±29 |
| PFR | 450±36 | 88±29 |
| HCO_3_^-^ [mEq/L] | 26±3 | 22.7±6.0 |
| FiO_2_ | 0.5 | 1.0 |

**Table E1.** *Definition of abbreviations:* pH = arterial pH; PCO_2_ = partial pressure of carbon dioxide, expressed in millimeters of mercury; PO_2_ = partial pressure of oxygen, expressed in millimeters of mercury; PFR = PaO_2_ to FiO_2_ ratio; HCO_3_^-^ = arterial bicarbonate ion concentration, expressed in milliequivalents per Liter; FiO_2_ = fraction of inspired oxygen. Baseline = values recorded in sheep with healthy lungs; ARDS = values recorded after the induction of experimental ARDS via intravenous oleic acid injection.

**Additional References**

**E1.** Sousa SB, Ferreira C, Silva JS, Silva A, Teixeira L**: Quantitative evaluation of a pulmonary contour segmentation algorithm in X-ray computed tomography images.** *Acad Radiol* 2004, 11:868-878.
